# No evidence of critical slowing down in two endangered Hawaiian honeycreepers

**DOI:** 10.1371/journal.pone.0187518

**Published:** 2017-11-13

**Authors:** Jessica C. Rozek, Richard J. Camp, J. Michael Reed

**Affiliations:** 1 Department of Biology, Tufts University, Medford, MA, United States of America; 2 Hawai`i Cooperative Studies Unit, University of Hawai`i at Hilo, Hawai`i National Park, United States of America; 3 Pacific Island Ecosystems Research Center, U.S. Geological Survey, Hawai`i National Park, HI, United States of America; Auburn University, UNITED STATES

## Abstract

There is debate about the current population trends and predicted short-term fates of the endangered forest birds, Hawai`i Creeper (*Loxops mana*) and Hawai`i `Ākepa (*L*. *coccineus*). Using long-term population size estimates, some studies report forest bird populations as stable or increasing, while other studies report signs of population decline or impending extinction associated with introduced Japanese White-eye (*Zosterops japonicus*) increase. Reliable predictors of impending population collapse, well before the collapse begins, have been reported in simulations and microcosm experiments. In these studies, statistical indicators of critical slowing down, a phenomenon characterized by longer recovery rates after population size perturbation, are reported to be early warning signals of an impending regime shift observable prior to the tipping point. While the conservation applications of these metrics are commonly discussed, early warning signal detection methods are rarely applied to population size data from natural populations, so their efficacy and utility in species management remain unclear. We evaluated two time series of state-space abundance estimates (1987–2012) from Hakalau Forest National Wildlife Refuge, Hawai`i to test for evidence of early warning signals of impending population collapse for the Hawai`i Creeper and Hawai`i `Ākepa. We looked for signals throughout the time series, and prior to 2000, when white-eye abundance began increasing. We found no evidence for either species of increasing variance, autocorrelation, or skewness, which are commonly reported early warning signals. We calculated linear rather than ordinary skewness because the latter is biased, particularly for small sample sizes. Furthermore, we identified break-points in trends over time for both endangered species, indicating shifts in slopes away from strongly increasing trends, but they were only weakly supported by Bayesian change-point analyses (i.e., no step-wise changes in abundance). The break-point and change-point test results, in addition to the early warning signal analyses, support that the two populations do not appear to show signs of critical slowing down or decline.

## Introduction

Although the Hawaiian Islands have been a center of rapid and expansive species radiation [1,2], they are also a hotspot of species endangerment and extinction [3,4]. At least one-third of the bird species currently listed under the U.S. Endangered Species Act (USESA) are endemic to Hawai`i, including 16 species of honeycreeper [5,6]. Following colonization by Polynesians less than 800 years ago [7] and before first Western contact in 1778, more than half of the islands’ endemic avifauna—55 species—went extinct, and another 19 species disappeared after the arrival of Europeans [8,9]. An additional nine species currently listed as endangered under the USESA are likely extinct [10,11]. Current threats to endangered Hawaiian birds include habitat loss and degradation, introduced competitors and predators, and introduced diseases such as avian malaria and pox [5, 12–15]. Extinction risk is expected to be exacerbated by climate change through increasing habitat degradation from exotic, invasive plants and increasing altitudinal limits of disease [16–19].

We are specifically interested in the current status and extinction risks of the Hawai`i Creeper (*Loxops mana*) and the Hawai`i `Ākepa (*L*. *coccineus*), endangered insectivorous honeycreepers endemic to the island of Hawai`i. Population size of Hawai`i Creepers are stable or increasing in Hakalau Forest National Wildlife Refuge, but outside the refuge numbers have declined since the late 1970s [10]. Hawai`i `Ākepa range and abundance changes over recent decades are less clear, with some evidence of range contraction, densities increasing between the 1970s and 1990s but apparently decreasing subsequently [10]. Hakalau Forest National Wildlife Refuge, island of Hawai`i (hereafter, Hakalau) was established in 1985 explicitly to protect native Hawaiian forest birds, after comprehensive surveys in the late 1970s and early 1980s [20] identified that the largest populations of forest birds were found outside protected areas. Since its creation, Hakalau has had a history of active management including reforestation from cattle pasture, control of invasive plants, eradication of feral cattle and pigs, and long-term bird monitoring [21], making it an important study site for Hawaiian forest bird protection and restoration [22]. However, at Hakalau, there is controversy regarding the current status and trends, and therefore short-term extinction risks, of our two target species, the creeper and the `ākepa. The grounds for the argument come from two data sources. The first is the long-term abundance data from 1987 to 2012 across the Hakalau region. Camp et al. [22–24] have used increasingly sophisticated regression analyses on these time series, and consistently report long-term trends of these species to be either increasing (creeper) or inconclusive (neither clearly increasing nor declining; `ākepa). In contrast, Freed and Cann [25, 26] argue that piecewise regression analyses with a break-point (a point in a time series beyond which the slope of the relationship changes significantly) at the year 2000 suggests the species have started to decline. The year 2000 coincides with the start of a population surge of the Japanese White-eye (*Zosterops japonicus*), an introduced, invasive avian competitor [25, 27]. However, Camp et al. [23] found only weak statistical support for change-points (where mean values on either side of the point are statistically, significantly different) or break-points when analyzing the entire time-series and none of the change-points occurred in 2000. The other source of data used by Freed et al. as evidence that white-eyes pose a significant threat to creepers and `ākepas comes from local studies of factors such as parasite loads, molt patterns, and chick growth and mortality [27, 28]. For example, they report an unprecedented increase of ectoparasites starting in 2003 that may be due to horizontal transfer from introduced host birds and in native birds. Freed et al. [29] report parasite loads to be correlated with signs of nutritive stress, such as major fault bars on the wing and tail.

If there is a recent or impending transition in creeper and `ākepa populations, from persistent dynamics to dynamics associated with imminent decline, it might be statistically detectable using recently developed early warning signals of critical transition. This type of transition is characterized by a rapid shift in a non-linear system (e.g., population or ecosystem) between alternate stable states [30, 31]. Of particular interest here are signals associated with critical slowing down, a phenomenon characterized by longer recovery rates after population size perturbation [32–35], that might be detectable in a time series of population sizes. For such populations, commonly reported early warning signals of an impending shift from a trajectory of persistence to an extinction (or declining) trajectory include increases in autocorrelation, variance, coefficient of variation, skewness, and recovery time from a perturbation [31, 32, 35]. These types of transitions between stable states are expected in populations exhibiting sigmoidal population growth, which is caused by density-dependence in population growth. Theory based explanations of critical slowing down focus on the existence in these systems of transcritical or fold bifurcations [36–38]. Oddly, even though many of the statistical measures of critical slowing down are mathematically related [39], most published research reports little consistency between signals within a study (e.g., [40, 41]). Despite this, early warning statistical tools are being promoted for their potential benefit in species conservation and the most robust signal appears to be increasing autocollinearity [40, 42]. Most research to date on early warning signals of transition between persistence and a declining trajectory derived from a time series of population size or density has been in simulation (e.g., [40, 43]) or microcosm experiments (e.g., [37, 41]). Until recently, there have been relatively few applications of early warning signals to real populations (but see [44–48]). Results of these and other studies show mixed results in detecting early warning signals of impending population collapse (e.g., [49, 50]). Despite a growing number of simulation and microcosm studies of early warning signals in population size time series, there are no rules-of-thumb yet available for how much change in an early warning signal is sufficient to cause a resource manager to become concerned and enact alternative management activities [51].

Here, we evaluate 25-year (1987–2012) time series for the creeper and `ākepa from Hakalau using detection-corrected, state-space abundance estimates (from [24]) to look for evidence of early warning signals of impending population collapse related to the increase in white-eye abundance that started in 2000. If a recent or impending transition in population trajectory exists, we may be able to detect it by evaluating both time series for evidence of three early warning signals of critical slowing down: increased autocorrelation, increased variance, and increased linear skewness (L-skewness). For the purposes of this study, we looked for consistent increases in the selected early warning signals prior to and following 2000, when the creeper and `ākepa populations are argued to have transitioned from persisting to initiating biologically significant declines [27–29]. Finally, we looked for evidence of break-points in the detection-corrected, state-space abundance estimates, as they might be indicators of subtler population changes not meeting the criteria for a critical transition.

## Methods

### Species and study area

**Fig 1:**
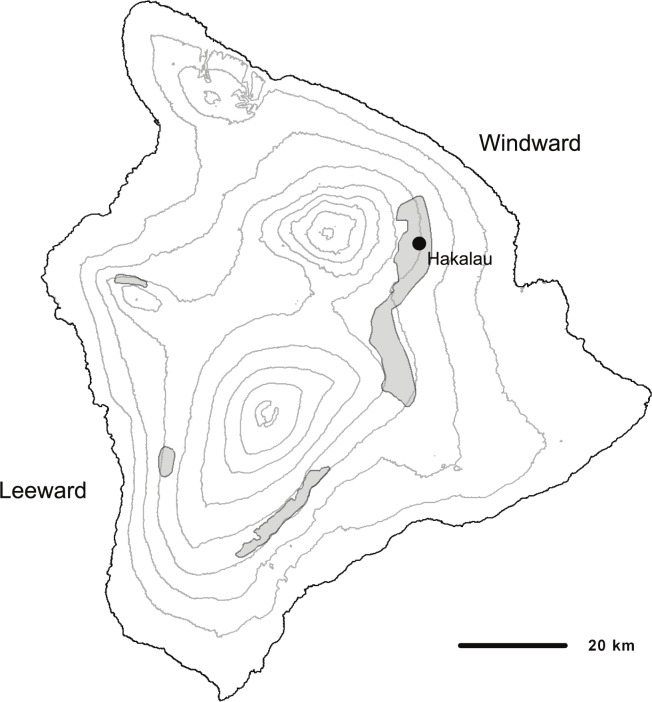
Estimated range of the Hawai`i Creeper (gray shaded areas) and approximate location of Hakalau on Hawai`i (courtesy of M. Goressen, HCSU, University of Hawai`i at Hilo).

**Fig 2:**
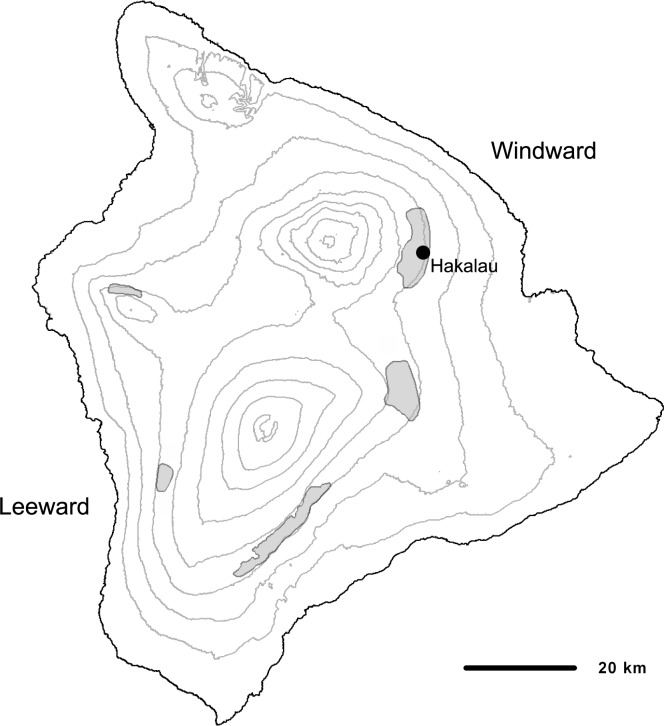
Estimated range of the Hawai`i Ākepa (gray shaded areas) and approximate location of Hakalau on Hawai`i (courtesy of M. Goressen, HCSU, University of Hawai`i at Hilo).

The creeper and `ākepa are endemic to the island of Hawai`i and found in ‘ōhi`a (*Metrosideros polymorpha)* and ‘ōhi`a-koa (*Acacia koa)* forests above 1500 m [20]. The creeper is a small greenish bark-gleaner that searches for insects along and under bark primarily on branches of ‘ōhi`a and koa [52]. It is found in four separate populations located in windward regions across the island with highest densities occurring in areas least affected by logging and grazing ([10, 20]; Fig 1). The `ākepa is also divided into similarly disjunct populations across the island ([10]; Fig 2). This brightly colored bird has a higher degree of specialization in feeding ecology than does the creeper [9]. The `ākepa uses its asymmetric bill to open leaf and flower buds while foraging for insects on koa and ‘ōhi`a (Lepson and Freed 1997, Pratt 2005). Both birds have low annual reproductive output compared to similar sized, continental passerines, usually laying 1–2 eggs in a single nesting attempt each year [15, 53]. According to the Hawai`i Forest Bird Survey, in the 1970s, population estimates for the creeper and the `ākepa across the island of Hawai`i were approximately 12,500 and 14,000 birds, respectively [20]. Gorresen et al. [10] estimated that creepers have increased to about 14,000 birds with densities increasing in Hakalau and possibly stable in upper Ka`ū, but decreasing in the central windward portion and nearly extirpated on the leeward side of Hawai`i Island. Gorresen et al. [10] estimate that the `ākepa population has declined since the 1970s to about 12,000 birds, with similar population patterns as those of the creeper–increasing in Hakalau, possibly stable in upper Ka`ū, decreasing in central windward, and nearly extirpated on leeward Hawai`i Island. Substantial populations of both species are found in Hakalau [10].

Abundance estimates of the creeper, `ākepa, and white-eye used in this study were generated from a 25-year (1987–2012) annual sampling effort in Hakalau ([24]; open forest stratum, their Table S4). The open canopy forest has 204 survey stations (184 ± 21 surveyed annually) spaced 150–250 m apart along transects spaced 500–1,000 m apart [22]. Population abundance estimates from raw counts were produced using distance sampling techniques to correct for imperfect detection probabilities, which were then refined using state-space models [24].

### Early warning signal analyses

We detrended the state-space abundance estimates using the ‘pracma’ package [54] in R (R version 3.3.1; [55]) and the natural log transformed detrended data were used for early warning signal analyses. We analyzed three indicators of critical slowing down: autocorrelation at lag-1 was calculated in R using the ‘earlywarnings’ package [56], sample variance was calculated as $\frac{\sum {\left(x-\bar{x}\right)}^{2}}{\left(n-1\right)},$ and L-skewness was calculated per Hosking [57] and Hosking and Wallis [58]. We selected L-skewness, *sensu* Hosking [57, 59], rather than the ordinary third moment because the latter is biased, particularly for small samples (n≤100) [60]. The first three linear moments were calculated as (from the L-Moment Regional Analysis Program User’s Manual, http://www.mgsengr.com/LRAP/Download/LMoments.pdf):

Mean = L_1_
Linear CV (t_2_): $t_{2}=\frac{L_{2}}{L_{1}}$
Linear Skewness (t_3_): $t_{3}=\frac{L_{3}}{L_{2}}$

Where the probability weighted moments are:

L_1_ = β_0_
L_2_ = 2β_1_ − β_0_
L_3_ = 6β_2_ − 6β_1_ + β_0_

and, where the data (X_1:n_) are first ranked in ascending order from 1 to n and:

$\beta_{0}=n^{-1}\overset{n}{\underset{j=1}{\sum }}x_{j}$
$\beta_{1}=n^{-1}\overset{n}{\underset{j=2}{\sum }}x_{j}\left[\frac{\left(j-1\right)}{\left(n-1\right)}\right]$
$\beta_{2}=n^{-1}\overset{n}{\underset{j=3}{\sum }}x_{j}\left[\frac{\left(j-1)(j-2\right)}{\left(n-1)(n-2\right)}\right]$

Each putative early warning indicator needs to be calculated from sequential overlapping subsets of the data in a rolling window through the time series (i.e., values 1 to s, 2 to s+1, 3 to s+2, etc.). The length of the rolling window should be related to the response times of the system, where short windows may be appropriate for systems with fast timescales and long windows for slower timescales (Dakos, http://www.early-warning-signals.org/). There is an obvious tradeoff in selecting a window length in that the longer the window length the more reliable the baseline estimates, though this results in fewer data points to analyze, and increases the likelihood of missing a transition window. Drake and Griffen [37], in their study of *Daphnia* microcosms, selected a window of 8 days (in a study with daily surveys for 416 days); in contrast, it is not uncommon to use half of the length of a time series as the window size [42, 56]. For our analyses we calculated values for two rolling window lengths within each species’ time series: 50% of the time series (13-year window length) and 25% (6-year window length). Because ordinary skewness is commonly used as an early warning signal, possibly because its bias is unknown in this research field, we also calculated ordinary skewness using the ‘earlywarnings’ package in R to evaluate the differences between L-moment and ordinary moment estimates.

Early warning signal analyses were completed for the creeper and the `ākepa. We did not conduct an early warning signal analysis for the white-eye as the increase in their abundance is not disputed. We evaluated species-specific early warning signals for trends using a rolling window analysis of two lengths: 50% (which provided metrics for 1999–2012) and 25% (which provided metrics for 1992–2012). Because we are interested in evaluating the hypothesis of a critical transition in 2000, we also looked for a trend in each early warning signal prior to the year 2000 for the 25% rolling window analysis (1992–2000; “pre-2000 25%”). Kendall’s τ, a common quantitative measure of early warning signal trend, was calculated for each indicator in the three windows of interest: 50%, 25% and pre-2000 25%. Kendall’s τ indicates strong support of an increasing non-zero slope if values are near unity, and significant positive values are expected when a system is approaching a critical transition. In contrast, stable systems not approaching a critical transition are expected to exhibit no trend in early warning signals and thus have Kendall’s τ values near zero [49]. In addition, the slopes of the putative early warning signals can be calculated with linear regression, although the significance values will be biased low if the time series possess strong positive autocorrelation [47, 61]. To control for this bias, we evaluated the general trends over time of early warning signals using a generalized least-squares regression with serial correlation (AR(1)) model in the R package ‘nlme’ [62]. The slope of each early warning signal was assessed for its direction (positive or negative) and if its 95% confidence intervals bracketed zero. Early warning signal analyses were also conducted on the detection-corrected abundance estimates using the same methods as above.

### Population trends

Much of the controversy about the status and fate of the creeper and `ākepa come from conflicting results from trend assessments, so we reanalyzed the data using updated population estimates using additional statistical tools. Specifically, for each species (creeper, `ākepa, white-eye), we used two break-point tools and compared the resulting estimates to a Bayesian change-point analysis. Next, to determine biological significance of the break-points, we calculated the slopes on either side of estimated break-points and compared the 95% confidence intervals with a 25% rate of change over 25 years (full methods for Population Trends are in the Supporting Information, S1 Text). We were particularly interested in determining the changes in slope on either side of any break-points, as well as looking for evidence of step-wise changes in abundance (change-points) on either side of the year 2000, associated with the observed rapid population growth of white-eyes starting this year.

## Results

### Early warning signal analyses

**Fig 3:**
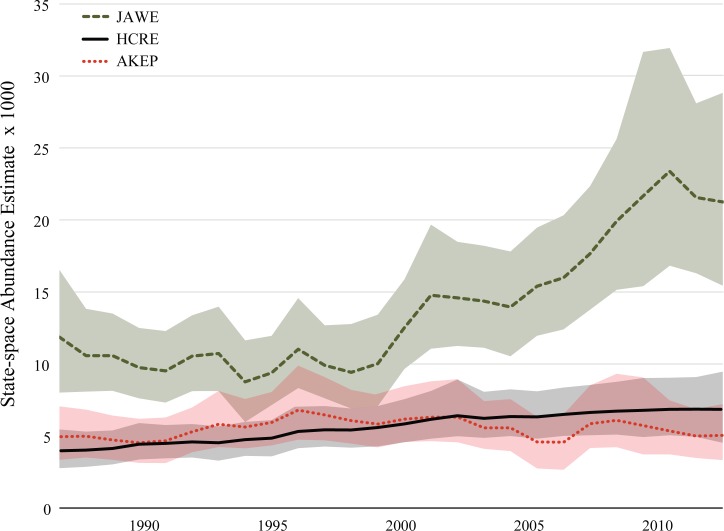
State-space abundance estimates (± 95% credible interval) of the Hawai`i Creeper (HCRE, black solid line), Hawai`i `Ākepa (AKEP, red dotted line) and the Japanese White-eye (JAWE, green dashed line) over 25 years (1987–2012) in the open forest stratum of Hakalau Forest National Wildlife Refuge. Modified from Camp et al. (2016).

**Fig 4:**
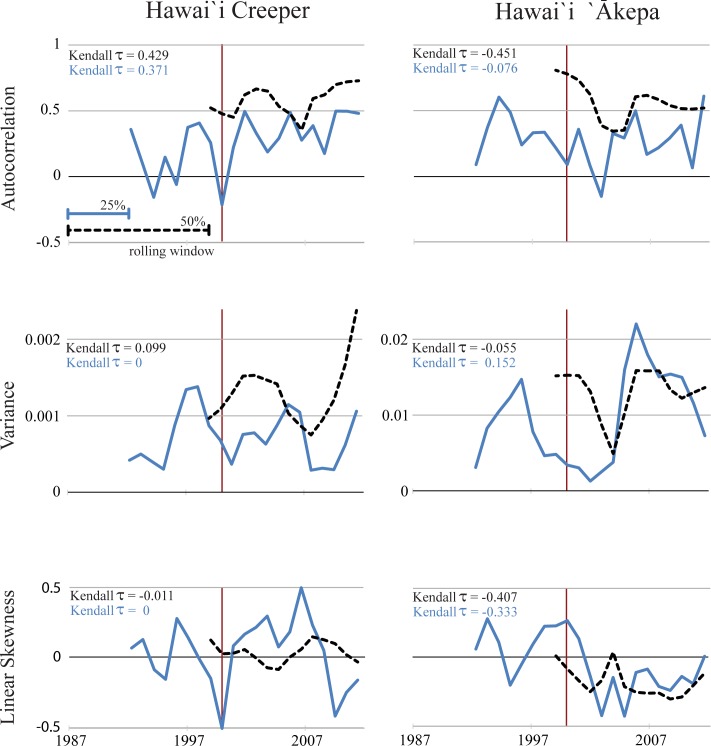
Autocorrelation, variance, and linear skewness with two rolling window sizes (complete 50% = black dashed line; complete 25% = blue solid line) for the Hawai`i Creeper and the Hawai`i`Ākepa. The vertical red line occurs at the year 2000, when the Japanese White-eye population started increasing (see Fig 3). Kendall’s τ indicates the strength of trend in indicators along the time series (complete 50% = black font, top; complete 25% = blue font, bottom).

State-space abundance estimates for the endangered creeper and `ākepa are relatively consistent over the 25-year period, while that of the introduced, invasive competitor, the white-eye, has a visually apparent increase starting in 2000 (Fig 3) and it is statistically supported, see below. However, early warning signal analyses for the creeper and the `ākepa revealed no evidence of critical slowing down. Specifically, there were no consistent increases in autocorrelation, variance, or L-skewness over the entire time series in either rolling window length, referred to as ‘complete 50%’ and ‘complete 25%’, or corresponding with approaching the year 2000, ‘pre-2000 25%’ (Fig 4). In our analyses, Kendall’s τ never exceeded 0.43 for any indicator in any window of interest suggesting that there are no strongly increasing trends in putative early warning metrics (Table 1). When evaluating the linear trends of the three early warning signals, all of the 95% confidence intervals of all slopes for each window of interest bracketed zero, except for autocorrelation in the total 25% window for the creeper. L-moment and ordinary moment calculations for skewness exhibited very different trajectories, sometimes changing in concert over time, while across other stretches of time they changed in opposite directions (S1 Fig). Neither metric, however, was associated in any clear way with time, prior to or following the year 2000. In addition, we found no evidence of critical slowing down when the same analyses were conducted on the detection-corrected abundance data estimates (S1 Table).

**Table 1:** Kendall’s τ and linear trends of the early warning signals autocorrelation, sample variance, and linear skewness in the Hawai`i Creeper and Hawai`i `Ākepa using state-space abundance estimates in Hakalau.

EWS metric	Kendall’s τ	Slope	95% CI	SE	T-value	P-value	Res. df	Assessment of slopes
**Hawai`i Creeper: 50% rolling window (1999–2012)**								
Autocorrelation	0.429	0.015	-0.011–0.041	0.013	1.12	0.28	12	No trend; confidence intervals of all slopes bracket zero
Variance	0.099	0.0001	-0.00004–0.0002	0.00008	1.37	0.19	12	
L-Skewness	-0.011	-0.012	-0.045–0.021	0.017	-0.71	0.49	12	
**Hawai`i Creeper: 25% rolling window (1992–2012)**								
Autocorrelation	0.371	0.018	0.004–0.031	0.007	2.51	0.02	19	Autocorrelation slope is positive; Variance and L-Skewness slope confidence intervals bracket zero
Variance	0	0.00002	-0.00003–0.00007	0.00003	0.56	0.57	19	
L-Skewness	0	-0.005	-0.037–0.026	0.016	-0.34	0.73	19	
**Hawai`i Creeper: Pre-2000 25% rolling window (1992–2000)**								
Autocorrelation	0	-0.059	-0.201–0.084	0.073	-0.80	0.44	7	No trend; confidence intervals of all slopes bracket zero
Variance	0.333	0.00003	-0.0002–0.0003	0.0001	0.26	0.79	7	
L-Skewness	-0.333	-0.072	-0.235–0.092	0.083	-0.85	0.41	7	
**Hawai`i `Ākepa: 50% rolling window (1999–2012)**								
Autocorrelation	-0.451	-0.022	-0.080–0.036	0.029	-0.75	0.46	12	No trend; confidence intervals of all slopes bracket zero
Variance	-0.055	-0.00009	-0.001–0.0009	0.0005	-0.17	0.86	12	
L-Skewness	-0.407	-0.011	-0.035–0.012	0.012	-0.95	0.35	12	
**Hawai`i `Ākepa: 25% rolling window (1992–2012)**								
Autocorrelation	-0.076	0.0003	-0.015–0.016	0.008	0.03	0.97	19	No trend; confidence intervals of all slopes bracket zero
Variance	0.152	0.0002	-0.001–0.002	0.001	0.32	0.74	19	
L-Skewness	-0.333	-0.013	-0.036–0.010	0.012	-1.12	0.27	19	
**Hawai`i `Ākepa: Pre-2000 25% rolling window (1992–2000)**								
Autocorrelation	-0.278	0.00001	-0.130–0.130	0.066	0.0002	0.99	7	No trend; confidence intervals of all slopes bracket zero
Variance	-0.167	0.00004	-0.003–0.003	0.001	0.02	0.97	7	
L-Skewness	0.333	0.024	-0.050–0.097	0.038	0.63	0.54	7	

### Population trends

The presence of some break-points were identified–that is, points where slopes changed. However, the years in which the break-points occurred differed by species and by statistical test used, and changes in means (Bayesian change-point tests) were at best weakly supported. Slopes before and after the break-points showed only minor changes, and these break-points were not associated with significant step-wise changes in abundance (change-point results; S2 Table, S2 Fig). The creeper population trend went from increasing before the break-point to less of an increase or no change after the break-point. The `ākepa transitioned from increasing to slight decline or no change. There was, however, a strongly supported (78%) break-point for the white-eye was identified in 1999, with population trends increasing after the break.

## Discussion

Statistical early warning signals of critical slowing down have been promoted as a potential tool for species conservation (e.g., [30, 40, 42]), though research on indicators from population size time series has rarely extended beyond simulation and microcosm experiments. In this study, we analyzed 25-year time series abundance estimates of two wild populations of endangered Hawaiian forest birds and did not find any support for a recent or impending transition to a declining population trajectory. Specifically, we looked for evidence of critical slowing down, associated with impending catastrophic transition in the creeper and the `ākepa in the open forest stratum in Hakalau, particularly around the year 2000, when white-eye numbers increased. We found no evidence of critical slowing down corresponding with the year 2000 or across the entire time series. The three early warning signal indicators analyzed, autocorrelation, variance and L-skewness, failed to show consistent increases over time in either rolling window length.

There are two scenarios that could yield our results: either we did not detect a signal because there was no change—or impending change—in state for either species and their current state is optimistic, or we failed to detect a signal that is present (a Type II error). There are conditions that could prohibit detecting a signal that is present, and some types of critical transitions do not exhibit critical slowing down (e.g., [38, 50, 63, 64]). One possibility is that early warning signals could be masked by environmental noise (e.g., annual stochastic variation in food availability) and by noise caused by observation error. For example, Perretti and Munch [51] found in population simulations with moderate additive noise that indicators failed to produce detectable signals of critical transitions more than half of the time (see also [42]). The advent of state-space models, as used in this study, should reduce this potential problem. In these models, each year’s estimate is informed by the previous year’s population size, and environmental and demographic variation is partitioned from observation error, making the estimates more dependable and representative of natural biological processes [24]. The state-space model provides the most accurate estimates as observation error from multiple sources (e.g., spatial distribution, temporary emigration) has been partitioned and eliminated. In the case of the `ākepa and the creeper, the detection-corrected abundance estimates had high estimated observation error (71% and 90%, respectively) which is not atypical for endangered species with low densities and heterogeneous distributions [24]. Detection-corrected estimates alone are unable to account for such sources of error, only imperfect detection. Since we know that detectability varies over time and has to be accounted for, it results in a two-stage analysis: 1) model detection probabilities and estimate abundances, and 2) evaluate trends in abundances using a state-space model. The detection-corrected, state-space model approach provides an accurate assessment of population status and trend. From these analyses we then evaluate the population pattern for early warning signals. Within the state-space model framework, the uncertainty within abundance estimates reduced the amount of modeled observation error. The process error among abundance estimates then reflects the population properties, and assessment using early warning indicator metrics should be useful to identify critical slowing down. We do note, however, that state-space models have the potential to over correct, leading to increased Type II error (i.e., critical slowing down has occurred but is not detected). It is also important to note that even if critical slowing down is rejected, small populations are still vulnerable to noise-induced state transitions, which do not exhibit early warning signals [39].

Another potential explanation for why we might not find evidence of critical slowing down, even if one were present, is that the duration of these time series may have been insufficient. Simulations and microcosm studies of very short-lived organisms can readily create statistically robust samples through replicate populations and relatively long time series, and researchers using these systems know the ultimate fates of their populations (e.g., [37]). In contrast, we were restricted to a single abundance-based time series for each species of only 25 years of annual surveys, where we do not know the near-future fate of the populations (persist, or go extinct). We note, however, that this is an impressive time series for monitoring terrestrial vertebrates, and better than what is available to many resource managers for other species. Although in theory (if not practically) the time series could have been increased by doing multiple surveys per year, this would not make biological sense because the species are seasonal, annual breeders. Therefore, a second or third survey each year would only parse the components of a pulsed event, rather than provide details of missed reproductive events. So, if the duration of this time series is insufficient, then early warning signals of impending state transition in population size might not serve the anticipated role in real-world conservation and management (e.g., [30]).

We think that it is important to also point out that even if we had found evidence consistent with early warning signals, that the result would need to be treated with caution. First, because of the possibility of a Type I error–a false positive signal–which will occur at a currently unknown rate. Second, because we are not certain about the mechanistic model underlying population growth, both a positive result and the assertion that it was driven by a particular mechanism would need to be treated as hypotheses to be further evaluated.

In addition to looking for evidence of critical slowing down, we also analyzed the time series using break-point and change-point tools to determine if other shifts in population trajectories were present despite the absence of early warning signals. We did this because it is part of the controversy central to our paper, and because a population with density-dependent growth could decline without exhibiting critical slowing down if it is declining due to reduced carrying capacity [65]. For our study species, habitat area has increased so reduced carrying capacity due to spatial constraints is not likely [22, 66], but carrying capacity could have declined due to increased competition (e.g., with white-eyes; [67]). There was strong support for a change-point in 1999 for the white-eye time series which corresponded with an increasing population trajectory. However, this increase in abundance did not coincide with any supported break-points in the time series of the creeper or `ākepa.

For both species, break-point tests using two methods yielded different results, and no break-points had corresponding change-points. In other words, no break-points and their consequent changes in slope were associated with a change in mean population abundance. Thus, we calculated the estimated slopes for the resulting piecewise regression for each detected break-point and compared them to thresholds defined by Camp et al. [22, 24]. If we assume that the break-points are real and evaluate slopes on either side of them, analyses indicated the creeper population has been increasing since 1987 and at the break-point (identified by different methods as 1999 and 2002) the rate of increase slowed. For the `ākepa, the results indicate the population was increasing until 1996 and has changed to a negligible/declining trend since then. Neither analysis categorically defined either species as having a definitive declining trend in abundance. It has been suggested that the white-eye invasion, which increased dramatically beginning in the year 2000, could drive the minor decrease in creeper population growth around this time [26]. Alternative explanations exist for slowing population growth. For example, the population could be approaching habitat-based carrying capacity, or a separate factor that caused creeper growth rate to decline also caused the subsequent increase (starting 4 years later) in white-eyes. The short-term increase in creeper and `ākepa populations between 1987 and their break-points might have been due to habitat quality improvement and carrying capacity increase after fencing and the removal of ungulates, particularly cattle and pigs, from some management units of Hakalau. The first management unit was fenced and ungulates removed in 1989, with more extensive work following in other units over the next 25 years [21, 68, 69]. We note that fence maintenance and other management activities waxed and waned during this time period, which may have influenced population trajectories since 1996 as the forest condition and carrying capacity fluctuated to varying degrees. Distinguishing these alternative hypotheses will be necessary to an ultimate resolution of the debate.

Some studies report the population trajectories of the creeper and the `ākepa as stable or increasing [22–24], while others report evidence of population decline or of impending collapse [25–29]. Kingsford [70] reviewed a subset of these studies and noted differences between them in survey methods, spatial and temporal scales, data analyzed, and statistical methods, with no clear way to reconcile the results. As an example, Kingsford [70] noted that Camp et al. [22] analyzed long-term abundance data from hundreds of survey stations across Hakalau starting in 1987 (open forest) and 1999 (closed forest), while Freed and Cann [25] analyzed various demographic measurements (e.g., banding, adult sex ratio, fledgling survival) that were collected in varying time intervals (1 to 18 years) at different sites across different years. We illustrate the differences in spatial and temporal scales between some of the studies involved in the controversy in S3 Fig. Potentially adding to the confusion, the dynamics of the forest have changed across the duration of the studies, with areas once classified as pasture gradually returning to forest [22, 66]. Consequently, one possible explanation for the disparities in population trajectories is that different events happening in different local sites across a subset of years were captured in Freed and Cann’s research, while the overall Hakalau-wide patterns were revealed in Camp et al.’s work and in our study (see also [71]). This might be analogous to observing a component (individual) Allee effect that does not result in a demographic (population) Allee effect [72]. That is, there can be reductions in individual reproductive success or survival (component Allee effect) that do not have population-level consequences (lack of demographic Allee effect). Consistent with this explanation, in some species, local habitats are known to drive reproduction, and thus micro-landscapes may influence demography [73]. In further support of this possibility, genetic population substructuring has been reported for island birds at very small spatial scales (10–20 km) [74, 75]. Finally, it could also be that neither group has a complete picture of the dynamics of the species because data come from different parts of the species’ ranges, or from different particular subsets of those ranges. A recent analysis of Christmas Bird Count data, where all survey circles occurred outside of Hakalau, proposed yet a different assessment: that the `ākepa experienced a population collapse starting around 1977 that ended around 1987 (though the authors note the population has recently hovered around their predefined collapse boundary), and the creeper experienced a collapse around 1998 and recovered around 2007 [76]. Although creeper and `ākepa populations within Hakalu appear to be rebounding, the species futures are by no means secure. Their population sizes are small, distributions restricted and possibly contracting, and subpopulations are isolated.

### Management implications

We found no support for early warning signals indicating a recent or impending critical slowing down in the populations of either species. It is not clear, however, how useful current early warning signals might be in anticipating critical transitions of natural populations. One stark difference between the application of early warning signal detection analyses to ongoing ecological data collection, as compared to simulation and microcosm studies, is that the fate of the study species (about to start a population crash or not) in a natural system is unknown. Simulation and microcosm studies make their assessments *post hoc*, while a resource manager needs to anticipate problems in species with unknown fates. In addition, early warning signal performance may be driver-dependent and multiple drivers acting simultaneously on a system, or system noise, might obscure or mask signals of critical slowing down [77]. What would benefit species conservation and ecosystem management is a set of sensitive, early warning indicators that work on the scale of time series data typical of managed systems. To achieve the desired goal of using these early warning signals in species management [30], however, rules-of-thumb and statistical criteria for the amount of change in signal that is associated with imminent collapse need to be developed or identified. Of concern in the application of early warning signals, Dakos et al. [56] suggested that there is no built-in way to test a null hypothesis for many of the rolling window metrics. In particular, resource managers need to know how much of a change in an indicator metric (or suite of metrics) is required before an alert limit is triggered and management actions are implemented to counter population declines. Without this, early warning signals of critical transitions are an interesting phenomenon with little practical application to conservation resource managers.

## Supporting information

S1 Text Methods and results for populations trends. (PDF)

S1 Table Early warning signal analysis of detection-corrected abundance estimates. Kendall’s τ and linear trends of the early warning signals autocorrelation, sample variance, and linear skewness in the Hawai`i Creeper and the Hawai`i `Ākepa using detection-corrected abundance estimates in Hakalau.
(PDF)

S2 Table Population trend results. Estimated break-points and piecewise regression slope estimates for the Hawai`i Creeper and the Hawai`i `Ākepa. Break-points are shown with the corresponding posterior probability (*P*) of a change-point at that time.
(PDF)

S1 Fig Linear skewness versus ordinary skewness. Linear skewness (black solid line) and ordinary skewness (brown dashed line) with two rolling window sizes (50% top graphs, 25% bottom graphs) for the Hawai`i Creeper and the Hawai`i `Ākepa. The vertical red line occurs at the year 2000, when the Japanese White-eye population increased and the purported start of both species’ population declines. Inset graphs (blue dots) depict the relationship between values of linear and ordinary skewness; abscissa is L-skewness and the ordinate is ordinary skewness.
(PDF)

S2 Fig Bayesian change-point analysis. Change-point assessment of the Hawai`i Creeper, Hawai`i `Ākepa, and Japanese White-eye natural log-transformed detection-corrected state-space generated abundance estimates from 1987 to 2012 in Hakalau using Bayesian change-point analysis (R package ‘bcp’). The upper plots show the annual abundance estimates (green dots) along with the posterior means at each annual survey (black solid, red dotted, and green dashed lines). The lower plots show the weight of the evidence for a change-point along the time series, as indicated by the Bayesian posterior probabilities (*P*). Included in the lower plots are the estimated break-point locations from R packages ‘segmented’ and ‘strucchange’ and their associated posterior probabilities of a change-point. As per Camp et al. (2010), we considered posterior probabilities <0.1 to be very weak; weak if 0.1≤ *P* < 0.5; moderately strong if 0.5 ≤ *P* < 0.7; strong if 0.7 ≤ *P* < 0.9; and very strong if *P* ≥ 0.9. Estimated break-points for the Hawai`i Creeper had weak (44%) and very weak (2%) support, the break-point estimated for the Hawai`i `Ākepa had very weak support (9%), and those fro the Japanese White-eye had very weak (2%) and strong (78%) support.
(PDF)

S3 Fig Comparison of methods of different research groups analyzing forest bird data in Hakalau. Comparison of the methods, and spatial and temporal scales described in six papers analyzing forest birds in Hakalau. Blue portions represent annual point-transect sampling (excluding 2009 in Camp et al. 2016 as indicated by the dashed line); green portions represent mist netting efforts; and red portions represent other methods, as described. Question marks on the timeline refer to unspecified dates (e.g., in Freed et al. 2008 the 1830m site is described as being sampled “during the mid-1990s and after 2002”). Dots refer to sampling efforts that occurred in a single year. “Unk. site” refers to sampling efforts not attributed to a specific area. In the case of the unknown sites in Freed and Cann 2013, a map illustrates the sites which may be at 1700 m and 1585 m based on a similar map (in Freed and Cann 2014) though it is still unclear which site was sampled during which years.
(PDF)
