# Supplementary material for: No evidence of critical slowing down in two endangered Hawaiian honeycreepers
Source: PLoS One. 2017 Nov 13;12(11):e0187518. doi: 10.1371/journal.pone.0187518 (PMC5683562; doi:10.1371/journal.pone.0187518)
Supplement: S1 Text — (PDF) [file pone.0187518.s001.pdf]

Population Trends

Methods

A variety of statistical tools are available to determine if and at what value break-points (the point at which a slope changes in a piecewise regression) or change-points (a step-wise change in mean) are present in a time series. The differences in break-point tools are numerable and may include the underlying algorithms, the objectivity of the estimated break-point parameters, the number of break-points included in the model, and whether or not the resulting piecewise regression is constrained to be continuous. Change-point tests can also vary in similar ways. Moreover, statistically significant break-point or change-point results may not always be ecologically relevant. In our analyses, we used two common break-point tools, one that constrains the regression lines to be continuous and one that does not. This is not the only difference between the packages; one requires a subjective starting point while the other does not, and there are differences in algorithms. The two packages, described below, were used to identify break-points in the detrended detection-corrected, state-space abundance data of the Hawai`i Creeper, Hawai`i `Ākepa and Japanese White-eye. We defined the resulting piecewise slopes as exhibiting an ecologically important decline or increase, or to exhibit negligible change based on predetermined threshold values (see below). We also compared the break-point estimates to the probability of a change-point to determine if the means between break-point segments are different as a change in slope does not necessarily lead to a change in mean population abundance. However, the value and importance of a break-point is not dependent on a change-point in the same location as a change in slope alone may be biologically meaningful.

First, we calculated 95% confidence intervals for break-points and trends using the R package 'segmented' (Muggeo 2008), which estimates break-points by iteratively fitting the linear model with a linear predictor. In this method, the broken-line relationship is constrained to intersect at the break-

point. In order to estimate a break-point, the algorithm must be provided with at least one initial subjective input as a starting point (psi) though this is not necessarily the resulting break-point estimate; thus we input the year 2000 that has been hypothesized to coincide with population changes. In our second break-point analysis, we used the R package 'strucchange' (Zeileis et al. 2002, 2003) which uses least squares regression via a dynamic grid search algorithm to estimate break-points based on Bai and Perron (2003a); the fitted lines are not required to join in this model. The model defaults of both R break-point packages were used.

We used a Bayesian analysis to test for change-points and their associated posterior probability (*P*) in each time series that exhibited a break-point with 1,000,000 iterations (and a burn-in of 50,000 iterations) using the R package 'bcp' (Erdman and Emerson 2007, 2008). A segment size of 50% of the time series was used as it has been argued that higher segment ratios may be more precise as they allow for different variances across segments and for serial correlation, and are less likely to lead to imprecise estimates and size distortions (Bai and Perron 2003b). As per Camp et al. (2010), we considered posterior probabilities < 0.1 to be very weak; weak if $0.1 \leq P < 0.5$; moderately strong if $0.5 \leq P < 0.7$; strong if $0.7 \leq P < 0.9$; and very strong if $P \geq 0.9$. Finally, to determine biological significance of estimated slopes before and after the break-points, we compared 95% confidence intervals of slopes against thresholds defined by a 25% rate of change over 25 years, as used by Camp et al. (2010, 2016): declining (< -0.0119); negligible (-0.0119 to 0.0093); or increasing (> 0.0093).

Results

Both the 'segmented' and 'strucchange' packages identified a break-point in the Hawai`i Creeper time series, although not in the same year: 2002 (95% CI: ± 2.6 years) and 1999 (95% CI: 1998 to 2001), respectively (the difference in confidence interval presentation is due differences in the package outputs). The assessment of the break-point in both packages based on the posterior probability

of a change-point, however, was that there was at most weak support for the break (2% for 2002 and 44% for 1999; S2 Table, S2 Fig). Piecewise regression showed similar patterns between the two packages with a change from an increasing trend until 2002 or 1999 to one that was increasing at a slower rate after the break-points (S2 Table). When comparing the 95% confidence interval of the slopes to biologically meaningful values, the trajectory of the first slope was identified as increasing while the second slope was identified as negligible/increasing (S2 Table). For the Hawai`i `Ākepa time series, the 'segmented' analysis found a break-point in 1996 (95% CI: ± 2.97 years) with very weak support (9%; S2 Table, S2 Fig). The 'strucchange' package failed to identify a break-point. Based on piecewise regression, the biological assessment of the change in slopes around this break-point was a shift from an increasing trajectory to one that bracketed the threshold between negligible change and declining (S2 Table). Both the 'segmented' and 'strucchange' packages identified a break-point in the Japanese White-eye time series, though, similar to the creeper, they were not in the same year: 1995 (95% CI: ± 1.79 years) and 1999 (95% CI: 1997 to 2000), respectively. Based on the posterior probability of a change-point, the break-point in 1995 had very weak support while the break-point in 1999 was strongly supported (2% and 78%, respectively; S2 Table, S2 Fig). Prior to both break-points, the population trajectory bracketed the threshold between declining and negligible change and after the break-points the slopes were increasing (S2 Table).

Supporting Information Literature Cited

Bai J., and P. Perron. 2003a. Computation and analysis of multiple structural change models. *Journal of Applied Econometrics* **18**:1-22.

Bai J., and P. Perron. 2003b. Critical values for multiple structural change tests. *The Econometrics Journal* **6**:72-78.

Camp R. J., T. K. Pratt, P. M. Gorresen, J. J. Jeffrey, and B. L. Woodworth. 2010. Population trends of forest birds at Hakalau Forest National Wildlife Refuge, Hawai'i. *The Condor* **112**:196-212.

Camp R. J., K. W. Brinck, P. M. Gorresen, and E. H. Paxton. 2016. Evaluating abundance and trends in a Hawaiian avian community using state-space analysis. *Bird Conservation International* **26**:225-242.

Erdman C., and J. W. Emerson. 2007. bcp: an R package for performing a Bayesian analysis of change point problems. *Journal of Statistical Software* **23**:1-13.

Erdman C., and J. W. Emerson. 2008. A fast Bayesian change point analysis for the segmentation of microarray data. *Bioinformatics* **24**:2143-2148.

Freed L. A., and R. L. Cann. 2009. Negative effects of an introduced bird on species growth and survival in a native bird community. *Current Biology* **20**:1736-1740.

Freed L. A., and R. L. Cann. 2010. Misleading trend analysis and decline of Hawaiian forest birds. *The Condor* **112**:213-221.

Freed L. A., and R. L. Cann. 2013. More misleading trend analysis of Hawaiian forest birds. *The Condor* **115**:442-447.

Freed L. A., and R.L. Cann. 2014. Diffuse competition can be reversed: a case history with birds in Hawaii. *Ecosphere* **5**: 1-40.

Freed L. A., R. L. Cann, and G. R. Bodner. 2008. Incipient extinction of a major population of the Hawai`i

Akepa owing to introduced species. *Evolutionary Ecology Research* **10**:931-965.

Muggeo V. M. 2008. Segmented: an R package to fit regression models with broken-line relationships. *R News* **8**:20-25.

Zeileis A., C. Kleiber, W. Krämer, and K. Hornik. 2003. Testing and dating of structural changes in practice. *Computational Statistics & Data Analysis* **44**:109-123.

Zeileis A., F. Leisch, K. Hornik, and C. Kleiber. 2002. Strucchange. An R package for testing for structural change in line.
