# Supplementary material for: No evidence of critical slowing down in two endangered Hawaiian honeycreepers
Source: PLoS One. 2017 Nov 13;12(11):e0187518. doi: 10.1371/journal.pone.0187518 (PMC5683562; doi:10.1371/journal.pone.0187518)
Supplement: S1 Table — Kendall’s τ and linear trends of the early warning signals autocorrelation, sample variance, and linear skewness in the Hawai`i Creeper and the Hawai`i `Ākepa using detection-corrected abundance estimates in Hakalau. (PDF) [file pone.0187518.s002.pdf]

S1 Table. Kendall's τ and linear trends of the early warning signals autocorrelation, sample variance, and linear skewness in the Hawai`i Creeper and Hawai`i `Ākepa using detection-corrected abundance estimates in Hakalau.

EWS metric	Kendall's τ	Slope	95% CI	SE	T-value	P-value	Res. df	Assessment of slopes
**Hawai`i Creeper: 50% rolling window (1999-2012)**								
Autocorrelation	0.604	0.021	0.013 – 0.027	0.004	5.88	0.0001	12	Autocorrelation slope is positive; Variance and L-Skewness slope confidence intervals bracket zero
Variance	-0.538	-0.005	-0.012 – 0.002	0.004	-1.31	0.21	12	
L-Skewness	0.552	0.007	-0.022 – 0.037	0.015	0.510	0.62	12	
**Hawai`i Creeper : 25% rolling window (1992-2012)**								
Autocorrelation	0.152	0.015	-0.025 – 0.055	0.021	0.75	0.46	19	No trend; confidence intervals of all slopes bracket zero
Variance	-0.514	-0.003	-0.016 – 0.011	0.007	-0.40	0.69	19	
L-Skewness	0.058	-0.014	-0.043 – 0.015	0.015	-0.94	0.36	19	
**Hawai`i Creeper: Pre-2000 25% rolling window (1992-2000)**								
Autocorrelation	0	0.003	-0042 – 0.048	0.023	0.14	0.89	7	No trend; confidence intervals of all slopes bracket zero
Variance	0.444	0.009	-0.012 – 0.030	0.011	0.86	0.42	7	
L-Skewness	-0.029	-0.008	-0.058 – 0.043	0.026	-0.30	0.78	7	
**Hawai`i `Ākepa: 50% rolling window (1999-2012)**								
Autocorrelation	-0.275	-0.020	-0.078 – 0.038	0.030	-0.68	0.51	12	L-Skewness slope is negative; Autocorrelation and Variance slope confidence intervals bracket zero
Variance	0.516	0.006	-0.001 – 0.014	0.004	1.60	0.14	12	
L-Skewness	-0.692	-0.027	-0.048 – -0.006	0.011	-2.49	0.03	12	
**Hawai`i `Ākepa: 25% rolling window (1992-2012)**								
Autocorrelation	-0.067	0.004	-0.023 – 0.031	0.014	0.28	0.79	19	L-Skewness slope is negative; Autocorrelation and Variance slope confidence intervals bracket zero
Variance	0.276	0.002	-0.020 – 0.023	0.011	0.18	0.86	19	
L-Skewness	-0.692	-0.039	-0.069 – -0.009	0.015	-2.52	0.02	19	
**Hawai`i `Ākepa: Pre-2000 25% rolling window (1992-2000)**								
Autocorrelation	-0.278	-0.020	-0.107– 0.068	0.045	-0.44	0.67	7	No trend; confidence intervals of all slopes bracket zero
Variance	-0.222	0.0003	-0.007 – 0.007	0.004	0.07	0.95	7	
L-Skewness	0.222	-0.098	-0.366 – 0.170	0.137	-0.71	0.50	7	
