# Supplementary material for: No evidence of critical slowing down in two endangered Hawaiian honeycreepers
Source: PLoS One. 2017 Nov 13;12(11):e0187518. doi: 10.1371/journal.pone.0187518 (PMC5683562; doi:10.1371/journal.pone.0187518)
Supplement: S2 Table — Estimated break-points and piecewise regression slope estimates for the Hawai`i Creeper and the Hawai`i `Ākepa. Break-points are shown with the corresponding posterior probability (P) of a change-point at that time. (PDF) [file pone.0187518.s003.pdf]

S2 Table. Estimated break-points and piecewise regression slope estimates for the Hawai`i Creeper and Hawai`i `Ākepa. Break-points are shown with the corresponding posterior probability (*P*) of a change-point at that time.

**Species, 'package': break-point year**	**Change-point *P***	**Slope Estimate**	**Lower 95% CI**	**Upper 95% CI**	**Bracket threshold?**	**Interpretation**
Hawai`i Creeper, 'segmented': 2002	2%					Very weak support
before, 1987-2002		0.0310	0.0288	0.0332	No	Increasing
after, 2002-2012		0.0109	0.0065	0.0154	Yes	Negligible/Increasing
Hawai`i Creeper, 'strucchange': 1999	44%					Weak support
before, 1987-1999		0.0295	0.0258	0.0332	No	Increasing
after 1999-2012		0.0120	0.0093	0.0148	Yes	Negligible/Increasing
Hawai`i `Ākepa, 'segmented': 1996	9%					Very weak support
before, 1987-1996		0.0351	0.0153	0.0550	No	Increasing
after, 1996-2012		-0.0137	-0.0234	-0.0038	Yes	Negligible/Declining
Japanese White-eye, 'segmented': 1995	2%					Very weak support
before, 1987-1995		-0.0233	-0.0460	-0.0053	Yes	Negligible/declining
after, 1995-2012		0.0555	0.0468	0.0642	No	Increasing
Japanese White-eye, 'strucchange': 1999	78%					Strong support
before, 1987-1999		-0.0093	-0.0216	0.0029	Yes	Negligible/declining
after, 1999-2012		0.0550	0.0426	0.0674	No	Increasing
