# Supplementary material for: No evidence of critical slowing down in two endangered Hawaiian honeycreepers
Source: PLoS One. 2017 Nov 13;12(11):e0187518. doi: 10.1371/journal.pone.0187518 (PMC5683562; doi:10.1371/journal.pone.0187518)
Supplement: S1 Fig — Linear skewness (black solid line) and ordinary skewness (brown dashed line) with two rolling window sizes (50% top graphs, 25% bottom graphs) for the Hawai`i Creeper and the Hawai`i `Ākepa. The vertical red line occurs at the year 2000, when the Japanese White-eye population increased and the purported start of both species’ population declines. Inset graphs (blue dots) depict the relationship between values of linear and ordinary skewness; abscissa is L-skewness and the ordinate is ordinary skewness. (PDF) [file pone.0187518.s004.pdf]

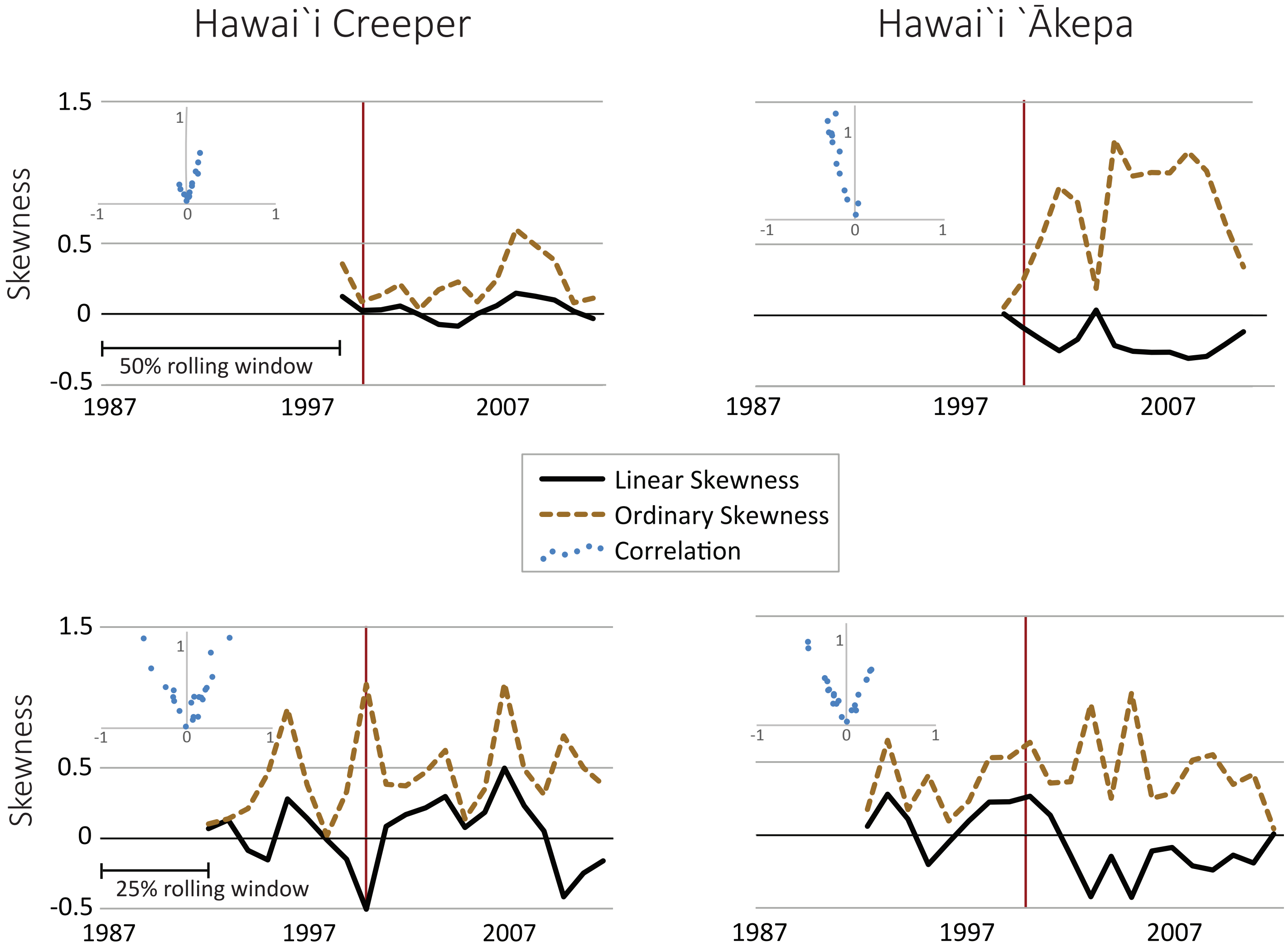

S1 Fig. Linear skewness (black solid line) and ordinary skewness (brown dashed line) with two rolling window sizes (50% top graphs, 25% bottom graphs) for the Hawai`i Creeper and the Hawai`i `Ākepa. The vertical red line occurs at the year 2000, when the Japanese White-eye population increased and the purported start of both species' population declines. Inset graphs (blue dots) depict the relationship between values of linear and ordinary skewness; abcissa is L-skewness and the ordinate is the skewness.
