# Supplementary material for: No evidence of critical slowing down in two endangered Hawaiian honeycreepers
Source: PLoS One. 2017 Nov 13;12(11):e0187518. doi: 10.1371/journal.pone.0187518 (PMC5683562; doi:10.1371/journal.pone.0187518)
Supplement: S2 Fig — Change-point assessment of the Hawai`i Creeper, Hawai`i `Ākepa, and Japanese White-eye natural log-transformed detection-corrected state-space generated abundance estimates from 1987 to 2012 in Hakalau using Bayesian change-point analysis (R package ‘bcp’). The upper plots show the annual abundance estimates (green dots) along with the posterior means at each annual survey (black solid, red dotted, and green dashed lines). The lower plots show the weight of the evidence for a change-point along the time series, as indicated by the Bayesian posterior probabilities (P). Included in the lower plots are the estimated break-point locations from R packages ‘segmented’ and ‘strucchange’ and their associated posterior probabilities of a change-point. As per Camp et al. (2010), we considered posterior probabilities <0.1 to be very weak; weak if 0.1≤ P < 0.5; moderately strong if 0.5 ≤ P < 0.7; strong if 0.7 ≤ P < 0.9; and very strong if P ≥ 0.9. Estimated break-points for the Hawai`i Creeper had weak (44%) and very weak (2%) support, the break-point estimated for the Hawai`i `Ākepa had very weak support (9%), and those fro the Japanese White-eye had very weak (2%) and strong (78%) support. (PDF) [file pone.0187518.s005.pdf]

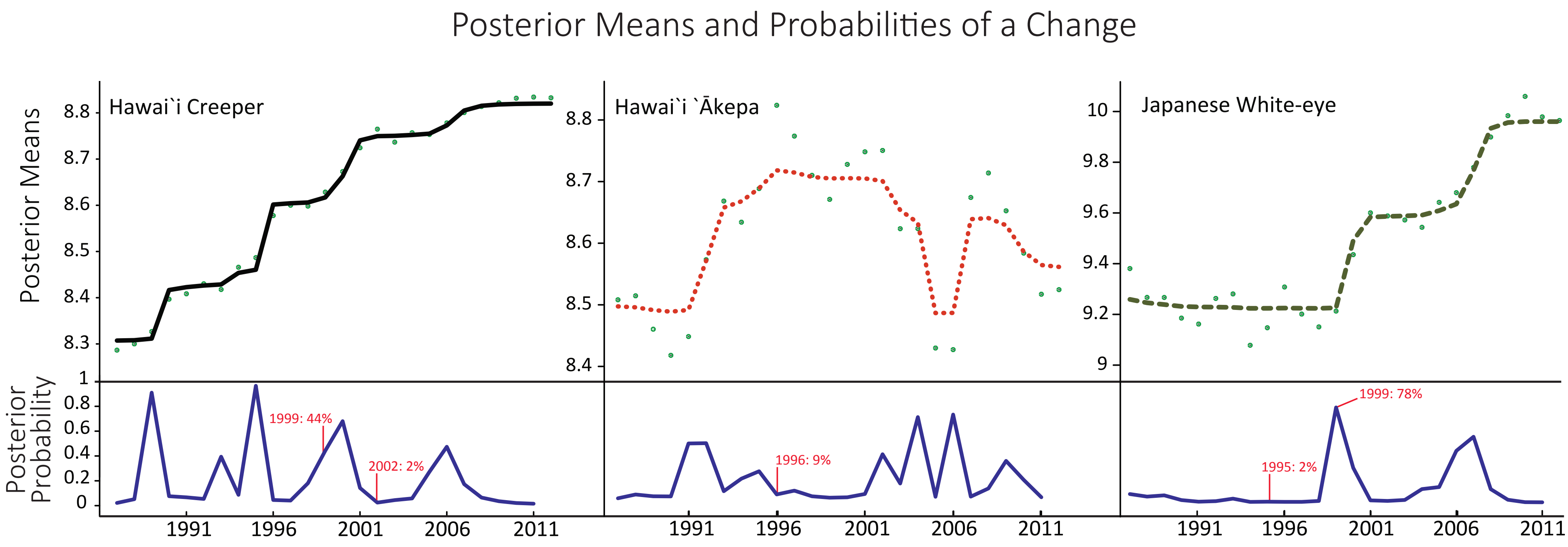

S2 Fig. Change-point assessment of Hawai`i Creeper, Hawai`i `Ākepa, and Japanese White-eye natural log transformed detection-corrected state-space generated abundance estimates from 1987 to 2012 in Hakalau using Bayesian change-point analysis (R package 'bcp'). The upper plots show the annual abundance estimates (green dots) along with the posterior mean at each annual survey (black solid, red dotted, and green dashed lines).The lower plots show the weight of evidence for a change-point along the time series, as indicated by the Bayesian posterior probabilities (P). Included in the lower plots are the estimated break-point locations from R packages 'segmented' and 'strucchange' and their associated posterior probabilites of a change-point. As per Camp et al. (2010), we considered posterior probabilieties < 0.1 to be very weak; weak if $0.1 \leq P < 0.5$; moderately strong if $0.5 \leq P < 0.7$; strong if $0.7 \leq P < 0.9$; and very strong if $P \geq 0.9$. Estimated break-points for the Hawai`i Creeper had weak (44%) and very weak (2%) support, the break-point estimated for the Hawai`i `Ākepa had very weak support (9%), and those for the Japanese White-eye had very weak (2%) and strong (78%) support.
