# Supplementary material for: No evidence of critical slowing down in two endangered Hawaiian honeycreepers
Source: PLoS One. 2017 Nov 13;12(11):e0187518. doi: 10.1371/journal.pone.0187518 (PMC5683562; doi:10.1371/journal.pone.0187518)
Supplement: S3 Fig — Comparison of the methods, and spatial and temporal scales described in six papers analyzing forest birds in Hakalau. Blue portions represent annual point-transect sampling (excluding 2009 in Camp et al. 2016 as indicated by the dashed line); green portions represent mist netting efforts; and red portions represent other methods, as described. Question marks on the timeline refer to unspecified dates (e.g., in Freed et al. 2008 the 1830m site is described as being sampled “during the mid-1990s and after 2002”). Dots refer to sampling efforts that occurred in a single year. “Unk. site” refers to sampling efforts not attributed to a specific area. In the case of the unknown sites in Freed and Cann 2013, a map illustrates the sites which may be at 1700 m and 1585 m based on a similar map (in Freed and Cann 2014) though it is still unclear which site was sampled during which years. (PDF) [file pone.0187518.s006.pdf]

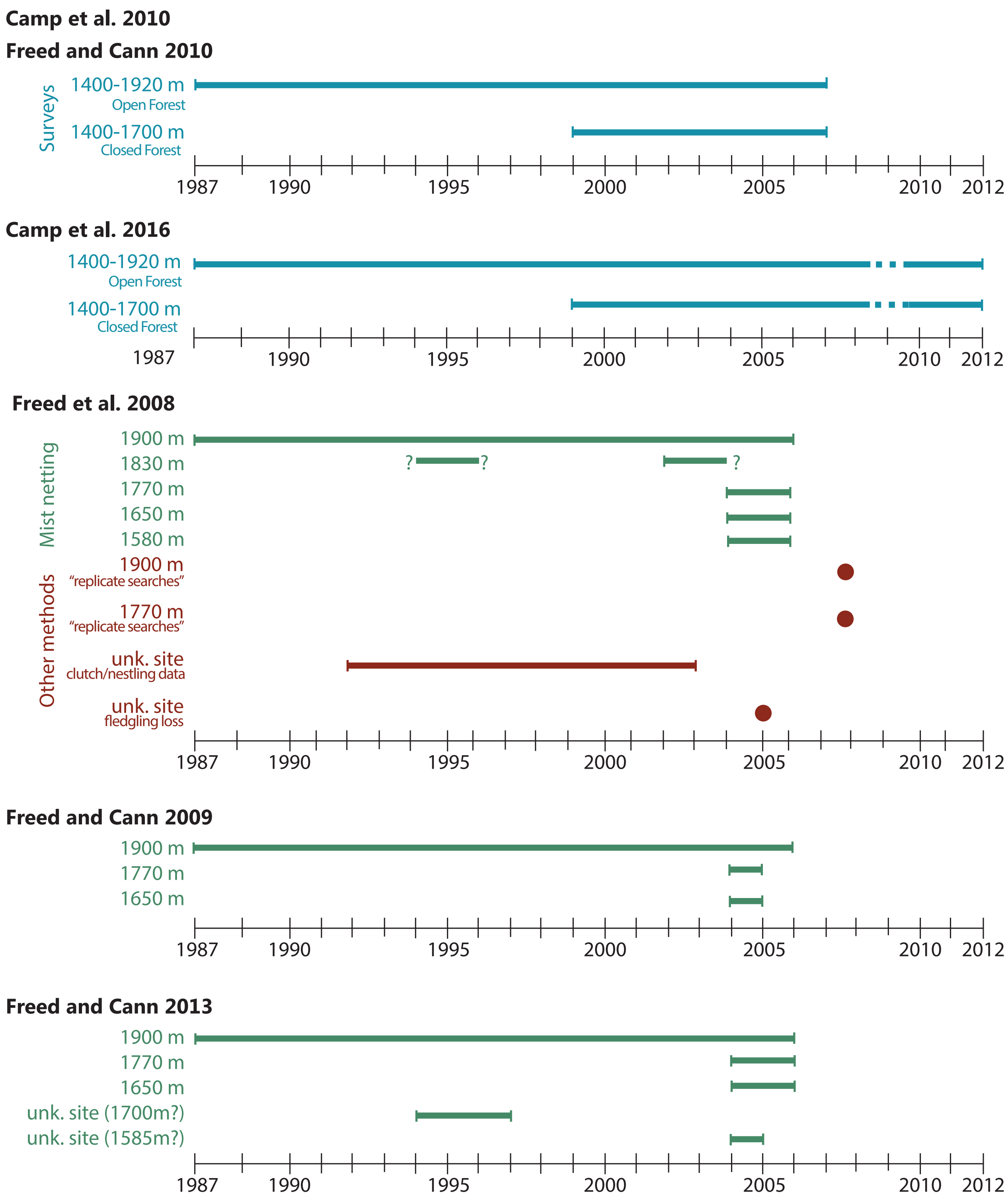

S3 Fig. Comparison of the methods, and spatial and temporal scales described in six papers analyzing forest birds in Hakalau. Blue portions represent annual point-transect sampling (excluding 2009 in Camp et al. 2016 as indicated by dashed line); green represents mist netting efforts; and red represents other methods, as described. Question marks on the timeline refer to unspecified dates (e.g., in Freed et al. 2008 the 1830m site is described as being sampled "during the mid-1990s and after 2002"). Dots refer to sampling effort that occurred in a single year. "Unk. site" refers to sampling efforts not attributed to a specific area. In the case of unknown sites in Freed and Cann 2013, a map illustrates the sites which may be at 1700 and 1585 m based on a similar map (Freed and Cann 2014) though it is still unclear which site was sampled during which years.
